# Transposable Element ‘*roo*’ Attaches to Nuclear Matrix of the *Drosophila melanogaster*

**DOI:** 10.1673/031.013.11101

**Published:** 2013-10-22

**Authors:** Anitha Mamillapalli, Rashmi U. Pathak, Hita S. Garapati, Rakesh K. Mishra

**Affiliations:** 1 Centre for Cellular and Molecular Biology, Uppal Road, Hyderabad-500 007, India; 2 Current Address: Department of Biotechnology, GITAM Institute of Science, GITAM University, Visakhapatnam-530 045, India

**Keywords:** genome organization, MAR DNA, retrotransposon

## Abstract

The genome of eukaryotes is organized into structural units of chromatin loops. This higher order organization is supported by a nuclear skeleton called the nuclear matrix. The genomic DNA associated with the nuclear matrix is called the matrix associated region (MAR). Only a few genome-wide screens have been attempted, although many studies have characterized locusspecific MAR DNA sequences. In this study, a MAR DNA library was prepared from the *Drosophila melanogaster* Meigen (Diptera: Drosophilidae) genome. One of the sequences identified as a MAR was from a long terminal repeat region of ‘*roo*’ retrotransposon (*roo* MAR). Sequence analysis of *roo* MAR showed its distribution across the *D. melanogaster* genome. *roo* MAR also showed high sequence similarity with a previously identified MAR in *Drosophila,* namely the ‘*gypsy*’ retrotransposon. Analysis of the genes flanking *roo* MAR insertions in the *Drosophila* genome showed that genes were co-ordinately expressed. The results from the present study in *D. melanogaster* suggest this sequence plays an important role in genome organization and function. The findings point to an evolutionary role of retrotransposons in shaping the genomic architecture of eukaryotes.

## Introduction

Chromatin in the eukaryotic nucleus is known to be organized into loop domains. Intranuclear space is compartmentalized into structural and functional domains (Spellman and Rubin 2002; Sexton et al. 2007; Kadauke et al. 2009; Cremer and Cremer 2010). The structural features of the nucleus are the nuclear membrane, nucleolus, and heterochromatic and euchromatic domains. The major functions involving chromatin, such as transcription, replication, repair, splicing, silencing, etc., are orchestrated in the non-chromatin space of the nucleus (Cook et al. 1999; Lanctot et al. 2007). The nuclear matrix (NuMat) has been proposed to play an important role in this structural and functional organization, as proteins related to the nuclear functions have been found to be physically associated with NuMat (Berezney and Wei 1998; Kallapagoudar et al. 2010).

Biochemically, NuMat is made of protein, RNA, and DNA. Protein and RNA constitute the bulk of NuMat, and only a small amount of DNA (∼1%) is found to be associated with it (Berezney and Coffey 1977). The DNA sequences associated with NuMat are called matrix-associated or scaffold-attachment regions (MARs/SARs). The MARs bind to NuMat and provide an anchor for higher order chromatin organization. This association is dynamic and varies in a cell-specific manner (Fey and Penman 1988; Dworetzky et al. 1990; Cai et al. 2003; Varma and Mishra 2011).

Earlier studies indicated that the association of MARs with NuMat leads to the formation of 50–200 kb chromatin loops that can act as independent functional domains (Jackson et al. 1990; Cremer and Cremer 2001). MAR DNA sequences range between 300 and 1000 bp in length and are AT rich (Boulikas 1993). These sequences were shown to have special sequence motifs, such as A-box (AATAAAAA/CAA) and T-box (TTTTATTTTT), and were also shown to bind to topoisomerase II, boundary element associated factor, and CCCTC-binding factor (CTCF) (Gasser and Laemmli 1986; Dunn et al. 2003; Pathak et al. 2007; Phillips et al. 2009). Many times they also coincided with replication origin (Amati and Gasser 1988). Though MARs contain specialized sequences, no consensus sequence motif had been identified before our study. It is presumed that the MAR property is determined by the structural similarities more than by the sequence similarity (Yamamura and Nomura 2001).

Computational programs that screen for genome wide occurrence of MAR sequences are far from perfect but they have useful predictive value (Evans et al. 2007). In the present study, a MAR DNA library from *Drosophila melanogaster* Meigen (Diptera: Drosophilidae) embryos was prepared. The long terminal repeat region (LTR) of transposable element ‘*roo’* was found as one of the MARs. Earlier studies have shown that a 350-bp sequence at the 5'-UTR of the *gypsy* transposon also had a nuclear matrix binding property (Nabirochkin et al. 1998). The sequence alignment of *roo* MAR with the NuMat associated region of *gypsy* showed very high similarity. Interestingly, a significant proportion of genes present in the flanking region of *roo* transposon were found to be expressed in adult testes and ovaries. These findings point to the importance of transposable elements in genome organization and evolution.

## Materials and Methods

### Isolation of MAR DNA of 0–16 hours old *Drosophila melanogaster* embryos

**Figure 1.**
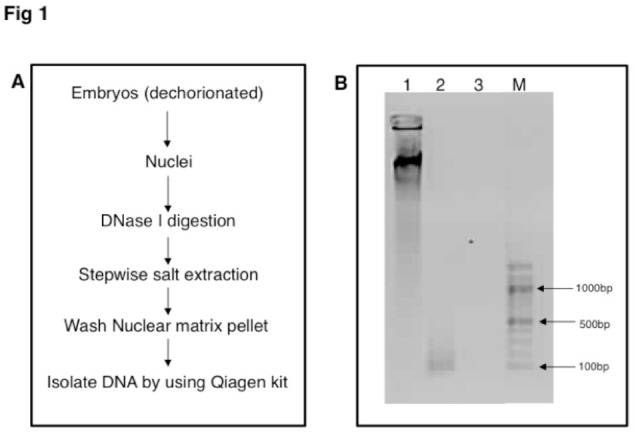
A: Flow chart of steps used for the isolation of MAR DNA from *Drosophila melanogaster* embryos. B: Ethidium bromide stained 1% agarose gel showing size distribution of MAR DNA from *D. melanogaster* embryos. Genomic DNA (lane 1); MAR DNA (lane 2); Isolated MAR DNA digested with DNase I (lane 3); 100 bp DNA marker (Lane M). High quality figures are available online.

Embryos (0–16 hrs old) were obtained from a laboratory population of *D. melanogaster* (Canton-S) maintained at 25° C. Embryos were collected and weighed. NuMat was prepared according to published protocol from 0.1 g of embryos (Mirkovitch et al. 1984) with modifications as mentioned in Pathak et al. (2007) (Figure 1). Briefly, nuclei were isolated in nuclear isolation buffer (15 mM Tris pH 7.4, 40 mM KCl, 1 mM EDTA, 0.1 mM EGTA, 0.1 mM PMSF, 0.25 mM spermidine, and 0.5% (v/v) Triton-X 100) with 0.25 M sucrose. The nuclear pellet was digested with digestion buffer (20 mM Tris pH 7.4, 20 mM KCl, 70 mM NaCl, 10 mM MgCl2, 0.125 mM spermidine, 1 mM PMSF, 0.5% Triton-X 100, 10 U/mL RNase In, and 40 U/µL DNase I) at 4° C for 1 hr to remove chromatin. Extraction was carried out sequentially with 0.4 M NaCl and then with 2.0 M NaCl, each for 5 min, in extraction buffer (10 mM Hepes pH7.5, 4 mM EDTA, 0.25 mM spermidine, 0.1 mM PMSF, 0.5% (v/v) Triton X-100). The final pellet after extraction was washed 2 times with wash buffer (5 mM Tris, 20 mM KCl, 1 mM EDTA, 0.25 mM spermidine, 0.1 mm PMSF), and DNA was isolated from the pellet using a DNeasy Blood and Tissue kit (Qiagen, www.qiagen.com).

### Preparation of MAR DNA library

The isolated MAR DNA was made blunt end with DNA polymerase I, large (Klenow) fragment (New England Biolabs, www.neb.com) and ligated to pMOS blunt end vector (Amersham kit, GE Healthcare, www.gelifesciences.com) according to the manufacturer's instructions. Transformed colonies were screened on blue-white selection and checked for inserts by restriction enzyme >digestions. DNA inserts in the plasmids were sequenced by the cycle sequencing method using the Big Dye terminator version 1.1 cycle sequencing kit (Applied Biosystems, www.appliedbiosystems.com) and an ABI Prism 310 Automated DNA sequencer (Applied Biosystems) with M13F and T7 primers.

### Analysis of library sequences

The library sequences were analyzed for MAR potential by MAR-WIZ program (Singh 2000) under the default parameters setting. The results are given in Table 1.

The MAR sequences were also analyzed for binding sites of DNA-binding proteins, such as boundary element associated factor, GAGA factor, zeste-white 5, suppressor of hairy wing, and dCTCF, using a bioinformatic tool known as “chromatin domain boundary element search tool — cdBEST” (Srinivasan and Mishra 2012). These proteins are know to interact with chromatin domain boundaries, and most of them have also been shown to bind with MARs. The results of the analysis are presented in Supplementary Table 1.

**Figure 2.**
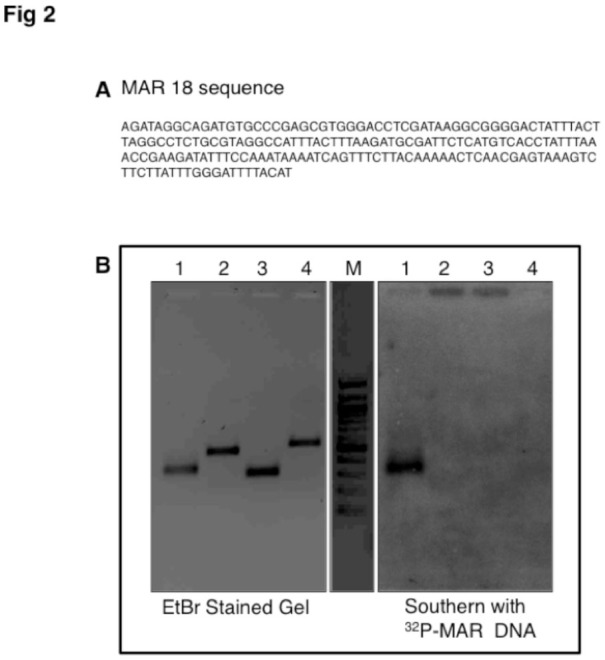
A: Sequence of MAR18 (*roo* transposon) clone found in MAR of *Drosophila melanogaster*. B: Southern blot analysis of PCR amplified *roo* LTR and control regions. Left panel shows the resolution of PCR amplicons on a 1.2% agarose gel. *roo* LTR (lane 1), Wnt4 control (lane 2), Arc control (lane 3), Wnt6 control (lane 4), 100 bp ladder (lane M). The right panel shows Southern hybridization of the gel with 32P-labelled MAR DNA. High quality figures are available online.

### Analysis of MAR18 (*roo* MAR) sequence

The library sequences were aligned with the *Drosophila* genome using NCBI-BLAST program (http://www.ncbi.nlm.nih.gov/). Of these, the MAR18 sequence was found to correspond to the LTR of *roo* transposon. Before proceeding further with any analysis, we first wanted to validate that the LTR of *roo* was actually associated with NuMat. To do this, an *in viv*o MAR assay was performed. Primers were designed to PCR amplify a region that enclosed the MAR18 sequence in the LTR of *roo* element (forward primer: 5′CCGCCTCCTAAAATAGTCCC3′; reverse primer: 5′CCTTACCTTTGGTAGGGGGA3′; amplicon size: 299 bp). As controls, primers were designed that amplified sequences of the *D. melanogaster* genome from an exon (in arc gene: forward primer: 5′GGAGAGGATTCAGGGTCACA3′; reverse primer: 5′GTTAGGGGAGGAGGAGCAAC3′; amplicon size: 280 bp), an intron (in Wnt6 gene: forward primer: 5′GAGAGACGGGTTTCGTGAAC3′; reverse primer: 5′CTTACCAATCGACCTGCGTT3′; amplicon size: 514 bp), or an intergenic region (5′ of Wnt4 gene: forward primer: 5′GATCTAGGCCGCATGGTAAA3′; reverse primer: 5′CGAGAGCTGAACCGAAAATC3′; amplicon size: 497 bp). These control fragments were from regions close to *roo* insertions. The amplicons were resolved on a 1.2% TAEagarose gel and transferred onto Nylon NY+ membrane in 20X SSC by capillary transfer. MAR DNA (obtained as mentioned above from *D. melanogaster* embryos) was labelled with 32P-dATP by the random primer labeling method. Hybridization was carried out at 60° C in 0.5 M sodium phosphate/7% SDS for 16 hr. The blot was washed stringently and exposed to a phosphor-imager screen for 4 hr. The results are presented in Figure 2.

**Figure 3.**
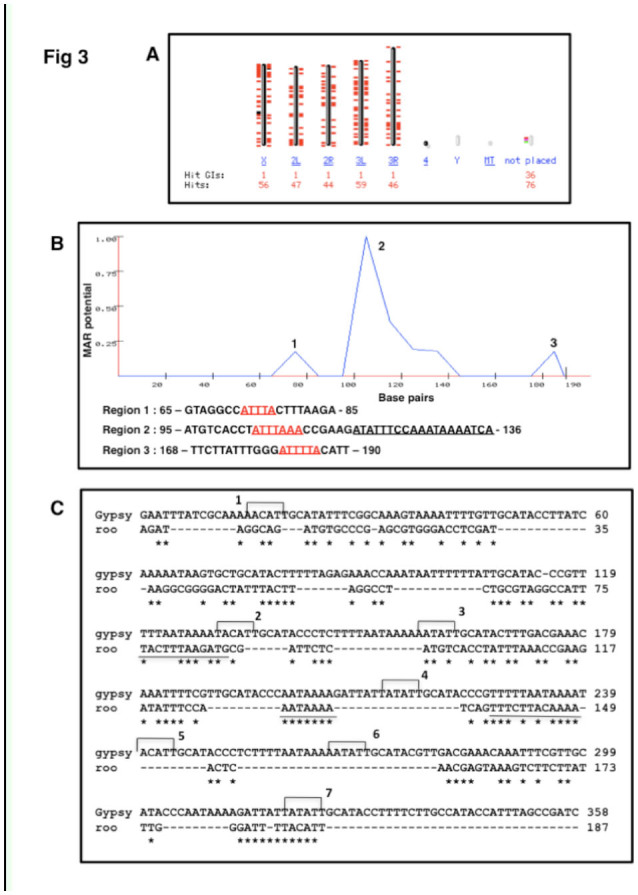
Analysis of *roo* MAR sequence. A: Genome view of distribution of *roo* MAR sequence in *Drosophila melanogaster*. B: Analysis of *roo* MAR with MAR-WIZ program. The regions with matrix association potential are shown as peaks in the graph. The matrix potential is shown on the Y-axis, and DNA in base pairs is shown on the X-axis. Sequences corresponding to the peaks are given below. Sequences relevant for MAR association are underlined. C: Sequence alignment of the *roo* MAR with the matrix-associated region of the *gypsy* transposable element using ClustalW program. On the *gypsy* sequence, topoisomerase II cleavage sites are marked with brackets and labelled 1–7. Sequences following ATC rule and an A-box are underlined. High quality figures are available online.

After validating that the *roo* LTR sequence was indeed retained in NuMat, *in silico* analysis of the transposon insertion sites in the *Drosophila* genome was performed. The NCBI-BLAST results were observed in a whole genome view. The 190 bp sequence was analyzed by MAR-WIZ to find out the sequences with high MAR potential. The *roo* MAR sequence was aligned to the previously identified MAR in *gypsy* transposon using CLUSTAL-W program (www.clustal.org). The results are presented in Figure 3.

### Analysis of genes that flank *roo* insertion sites in the *Drosophila* genome

**Figure 4.**
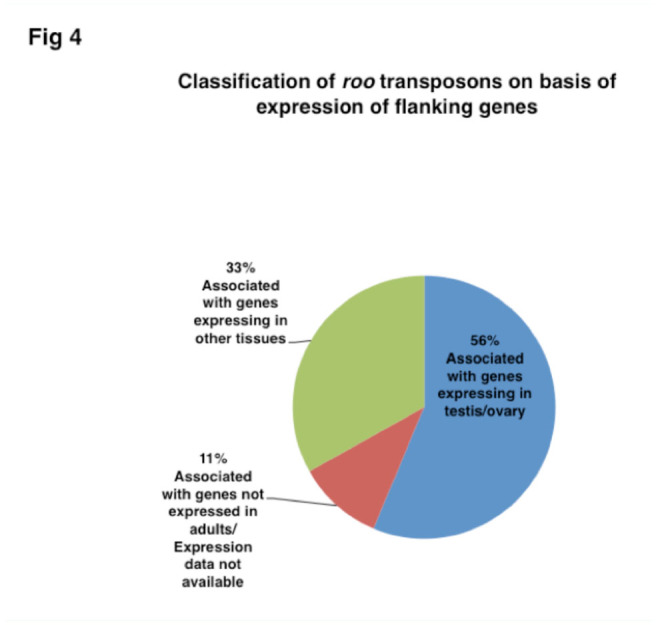
Pie chart showing classification of *roo* transposons from *Drosophila melanogaster* genome based on expression of flanking genes. High quality figures are available online.

The sequence locations of the *roo* transposon insertions in the whole genome of *D. melano gaster* were taken from FlyBase (www.flybase.org). The coordinates of the flanking genes were obtained from the release 5.45 of *D. melanogaster* available in FlyBase. The nearest genes associated with the *roo* transposons (upstream, downstream, and those containing them) were extracted using an inhouse written PERL script. For each of the associated genes, FlyAtlas anatomical expression data were obtained from FlyBase. The results are presented in Supplementary Tables 2 and 3 and Figure 4.

## Results

### Isolation of MAR DNA from *D. melanogaster* embryos

NuMat was prepared from 0–16 hr old *D. melanogaster* embryos using standard protocol (Figure 1A). Standard nuclear isolation protocols use hypertonic salt extraction to remove digested DNA. Alternative protocols using low salt extraction have been developed with the argument that physiological levels of salt may better preserve the ultrasturcture. However, a survey of literature shows that both methods reveal similar ultrastructural features (reviewed in Nickerson 2001). We used the high salt extraction method, modified so that the salt extraction was performed slowly in a step-wise manner (from low to high salt) in the presence of mild detergent. This ensured that the extraction process is gentle and avoids artifacts. From the NuMat pellet, MAR DNA was isolated. The size of MAR DNA ranged between 100 and 500 bp. Upon digestion of the isolated MAR DNA with DNase I, it was confirmed that the isolated fragments were DNA and not RNA (Figure 1B). The MAR DNA library was made according to the protocol described in the Methods. Despite repeated efforts, cloning did not give many colonies, probably because the MAR DNA were AT rich sequences with secondary structures. Such sequences are not tolerated well by the bacteria and hence are difficult to clone (Godiska et al. 2010; Leach and Lindsay 1986). The obtained MAR DNA clones were checked for inserts by restriction digestion. The size of the inserts ranged from 100 to 500 bp, correlating well with the size of the MAR DNA used for ligation. The clones were sequenced, and all the sequences obtained were found to be unique (Table 1).

### Analysis of the MAR DNA clones with MAR-WIZ and cdBEST programs

All the MAR clones were analyzed for the NuMat binding properties by *in silico* analysis. As no single property is attributed to NuMat association, we checked for AT%, origin of replication sites, topoisomerase II cleavage sites, AT richness (regularly spaced AT repeats), ATC rule (a stretch of 20 or more nucleotides of A, T, or C) ,and MAR score (all the individual parameters were considered, and those that had a potential higher than the threshold were given) with MARWIZ program (Singh 2000). Sixteen of the 35 sequences showed AT% of more than 60% (Table 1). Origin of replication sites were found in all the MAR sequences except 3. Two-thirds of the sequences showed AT richness. Sixteen sequences showed topoisomerase II sites. ATC rule was also followed by many of the clones, and most importantly all the clones showed maximum threshold for matrix association. All the sequences satisfied more than one rule of NuMat association. This analysis clearly indicated that the obtained sequences have potential to associate with NuMat, and the library represents a subset of the whole genome of MAR DNA sequences from *D. melanogaster* embryos.

The binding motifs of a few DNA binding proteins, such as boundary element associated factor, GAGA factor, zeste-white 5, dCTCF, and suppressor of hairy wing, were also checked for in the cloned sequences, as these proteins are reported to bind to chromatin domain boundaries as well as MAR sequences. Several boundaries have been shown to associate with NuMat, so whether any of the sequences had a potential for boundary activity was also checked. To check this, the cdBEST program (Srinivasan and Mishra 2012) was used. The program can be used for identification of recognition sequences of boundary interacting proteins as well as for identifying potential boundaries. The results (Supplementary Table 1) show that none of the MAR sequences cloned were predicted to be a potential boundary. Of the boundary/ MAR interacting proteins, the boundary element associated factor binding site was present in 10 sequences (∼29%), the GAGA factor binding site was present in 10 sequences (∼29%), and the zeste-white 5 binding site was present in 4 (∼10%) of the sequences. Although this data set is small, it indicates that all MAR sequences may not neccesarily act as boundaries and vice-versa. Further, MAR and boundary property, if present on the same sequence, may be separable and not overlapping.

### LTR sequence from *roo* transposon is enriched in NuMat

One of the clones from the library, labeled as MAR18, corresponded to an 190 bp sequence in the LTR of *roo* retrotransposon (Figure 2A). The complete *roo* retrotransposon element is 8.7 Kb, with a terminal repeat of 429 bp (Kaminker et al. 2002). The association of *roo* MAR with NuMat was validated by the *in vivo* MAR assay by Southern blotting. Primers were designed to amplify the LTR region of *roo* encompassing the MAR18 sequence. As controls, exonic, intronic, and intergenic regions close to *roo* insertion sites in the *Drosophila* genome were used. A signal in the *roo* MAR lane indicates the presence of complimentary sequences in the labelled MAR pool used as a probe. The absence of signals in the other lanes indicates that those sequences were not present in MAR *in situ* (Figure 2B). This experiment confirmed that the *roo* LTR element is associated with the NuMat *in vivo*.

### *In silico* analysis of *roo* MAR sequence

Upon BLAST analysis, *roo* MAR was shown to be present 250 times in the genome (56, 47, 44, 59, and 46 times on X, 2L, 2R, 3L, and 3R chromosomes respectively) (Figure 3A). *roo* MAR sequences were found both at intergenic and intronic regions but never in an exon. Sometimes it was present more than once within the same intronic or intergenic region. The sequence of *roo* MAR when analyzed using MAR-WIZ showed a region of maximum matrix association that extended from 95 bp to 135 bp of the LTR (Figure 3B). This region had an origin of replication sequence (ATTTA), a curved DNA sequence (TTTAAA), an A-box (AAATAAAA), and a region that conformed with ATC rule (underlined in the sequence). The other 2 regions with lower MAR potential also harbored origin of replication sequences and were AT rich. The sequence was further checked for its similarity with an already known MAR DNA sequence in *Drosophila gypsy* retrotransposon. Alignment showed overall 40–50% sequence similarity. In the *gypsy* MAR sequence, topoisomerase II recognition sites are labelled as 1 to 7, and regions showing ATC rule are underlined (Figure 3C) (Nabirochkin et al. 1998). The topoisomerase II recognition sequence numbered “7,” and the regions following ATC rule, showed high sequence conservation among *gypsy* and *roo* MAR. Furthermore, an A-box was present in both sequences. Thus, the 2 sequences were similar in regions important for MAR association.

### Analysis of *roo*-flanking genes in the *Drosophila* genome

FlyBase showed 193 insertions of *roo* in the whole genome of which 151 were in the sequenced region. Of the 151 places where *roo* transposon was inserted, 85 sites had a gene in the vicinity of those expressed in testes and ovaries (Supplementary Tables 2, 3), a significant 56% of the 151 sequenced *roo* insertions. Of the rest, expression data for genes around 11% of the *roo* insertions were either not available or the genes were not expressed in adult tissue. The remaining 33% insertions had associated genes expressed in other tissues (Figure 4). This analysis indicated a potential role for *roo* transposon in genome organization and regulated expression of distant genes via NuMat association.

## Discussion

The genome in eukaryotes needs MAR regions to demarcate chromatin into domains and to regulate gene expression (Heng et al. 2004; Razin et al. 2007). Many MARs have been characterized and are found to lie in genic as well as intergenic regions of the genome. MARs have been shown to topologically constrain DNA into loops. This plays an important role in compact packaging of the chromatin (Mirkovitch et al. 1984). As they are DNA sequences with special properties, several *in silico* programs attempt to predict these sequences on a genome-wide scale. MARs can target a DNA locus to a desired location for a specific function (Yusufzai and Felsenfeld 2004). For example, in *Drosophila*, the *scs'* boundary sequence that demarcates *hsp70* heat shock locus behaves as a MAR. It binds to the boundary element associated factor and localizes to the NuMat (Pathak et al. 2007). A similar example is *gypsy* retrotransposon, which is known to behave as an insulator. *Gypsy* DNA, along with its binding proteins, is located in the NuMat, and the intervening DNA between 2 *gypsy* insertions was found to be arranged in a loop (Byrd and Corces 2003). Mutation in the *gypsy* binding protein leads to disruption of the loop. In the context of spatial organization, such MARassociated localization could simply reflect changes in transcriptional status or changes in organization of chromatin structure.

In the present study, it was found that an abundant retrotransposon *roo* had a region that can bind to the NuMat. Transposon *roo* has been shown to be transcribed in a development and tissue-specific manner, and elements within the retrotransposon have been shown to act as *cis*-regulatory elements (Bronner et al. 1995). The transposon is distributed throughout the genome on all chromosomes. The genes flanking the transposon insertion site appeared to be coordinately regulated, as a sizable fraction of them were expressed in testes or ovaries. It would be ideal for the cell to have a few sequences and multiply them many times to organize the genome instead of having different sequences for different regions. These repeat sequences could provide the mechanism to identify coordinately regulated genes and cluster them in appropriate regions for regulated expression. Transposons like *roo*, by virtue of NuMat association, can act as a tool to direct the spatial organization of the genome and regulate expression. As they are mobile elements, they can lead to the creation of new domains by moving along the genome and helping in evolution. The findings of our study strengthen the idea of the role of mobile genetic elements in genome organization and gene regulation (Kazazian 2004; Tomilin 2008).

**Table 1.**
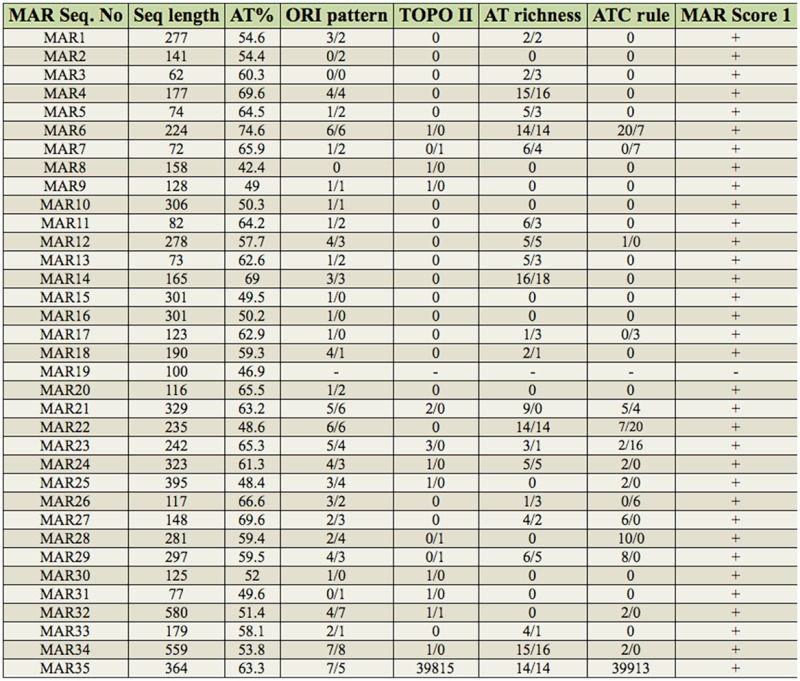
Characteristics of MAR DNA library sequences (Based on MAR-WIZ). Indvidual scores for origin of replication (ORI), Topoisomerase II (TopoII) sites, AT richness, and ATC rule are given for forward and reverse strands in F/R format.

## Figures and Tables

**Supplementary Table 1.**
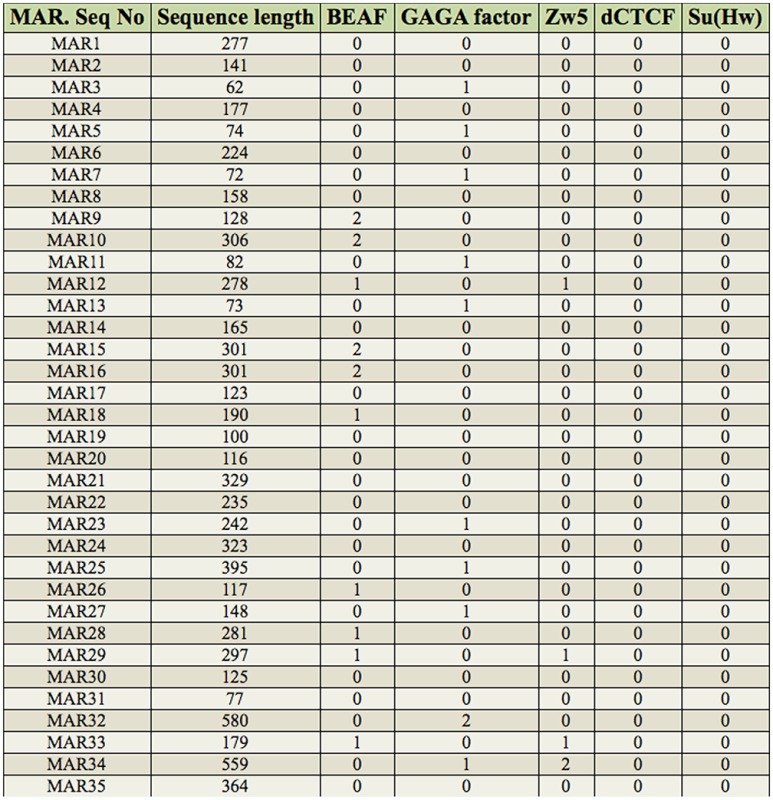
Binding sites for various boundary/MAR interacting proteins in the MAR DNA library sequences (Based on cdBEST).

## Abbreviations

CTCF,: CCCTC-binding factor;
LTR,: long terminal repeat;
MAR,: matrix associated region;
NuMat,: nuclear matrix
